# Neurotrophic Factors Protect the Intestinal Barrier from Rotavirus Insult in Mice

**DOI:** 10.1128/mBio.02834-19

**Published:** 2020-01-21

**Authors:** Marie Hagbom, Felipe Meira De Faria, Martin E. Winberg, Sonja Westerberg, Johan Nordgren, Sumit Sharma, Åsa V. Keita, Vesa Loitto, Karl-Eric Magnusson, Lennart Svensson

**Affiliations:** a Division of Molecular Virology, Department of Clinical and Experimental Medicine, Linköping University, Linköping, Sweden; b Division of Surgery, Orthopedics and Oncology, Department of Clinical and Experimental Medicine, Linköping University, Linköping, Sweden; c Division of Medical Microbiology, Department of Clinical and Experimental Medicine, Linköping University, Linköping, Sweden; d Division of Infectious Diseases, Department of Medicine, Karolinska Institute, Stockholm, Sweden; Indiana University Bloomington

**Keywords:** diarrhea, neurotrophic factors, permeability, rotavirus

## Abstract

Increased intestinal permeability has been proposed as a mechanism of rotavirus-induced diarrhea. Studies with humans and mice have, however, shown that rotavirus leaves intestinal permeability unaffected or even reduced during diarrhea, in contrast to most bacterial infections. Gastrointestinal permeability is regulated by the vagus nerve and the enteric nervous system, which is composed of neurons and enteric glial cells (EGCs). We investigated whether the vagus nerve, serotonin (5-HT), EGCs, and neurotropic factors contribute to maintaining gut barrier homeostasis during rotavirus infection. Using subdiaphragmatic vagotomized and 5-HT_3_ receptor knockout mice, we found that the unaffected epithelial barrier during rotavirus infection is independent of the vagus nerve but dependent on 5-HT signaling through enteric intrinsic 5-HT_3_ receptors. Immunofluorescence analysis showed that rotavirus-infected enterocytes were in close contact with EGCs and enteric neurons and that the glial cell-derived neurotrophic factor (GDNF) was strongly upregulated in enterocytes of infected mice. Moreover, rotavirus and 5-HT activated EGCs (*P < *0.001). Using Ussing chambers, we found that GDNF and *S*-nitrosoglutathione (GSNO) led to denser epithelial barriers in small intestinal resections from noninfected mice (*P < *0.01) and humans (*P < *0.001) and that permeability was unaffected in rotavirus-infected mice. GSNO made the epithelial barrier denser in Caco-2 cells by increasing the expression of the tight junction protein zona occludens 1 (*P < *0.001), resulting in reduced passage of fluorescein isothiocyanate dextran (*P < *0.05) in rotavirus-infected monolayers. This is the first report to show that neurotropic factors contribute to maintaining the gut epithelial barrier during viral insult.

## Abstract (precis)

Human and mouse studies have shown that rotavirus infection is associated with low inflammation and unaffected intestinal barrier at the time of diarrhea, properties different from most bacterial and inflammatory diseases of the gut. We showed by *in vitro*, *ex vivo*, and *in vivo* experiments that neurotrophic factors and 5-HT have barrier protective properties during rotavirus insult. These observations advance our understanding of how the gut barrier is protected against rotavirus and suggest that rotavirus affects the gut barrier differently from bacteria. This is the first report to show that neurotrophic factors contribute to maintain the gut epithelial barrier during viral insult.

## INTRODUCTION

Rotavirus infections are a leading cause of severe, dehydrating gastroenteritis in children under the age of 5 years. While rotavirus primarily infects intestinal enterocytes, the underlying mechanisms responsible for the diarrhea remain unresolved. Multiple mechanisms have been proposed, including rotavirus nonstructural protein 4 (NSP4) enterotoxin activity and activation of the enteric nervous system (ENS) (1–3). Alterations in intestinal permeability and therefore the ensuing potential electrolyte and water leakage as a mechanism of diarrhea have also been proposed and investigated *in vivo*. Such studies found that rotavirus does not alter intestinal permeability during diarrhea in humans (4–6) or mice (7), which is in contrast to the increased permeability observed during common enteric bacterial infections in humans (8, 9).

We have previously reported that rotavirus activates the ENS (10), stimulates serotonin (5-hydroxytryptamine [5-HT]) release from human enterochromaffin (EC) cells, and activates the nucleus of the solitary tract, part of the vomiting center, through vagus nerve signaling (11). These studies were recently extended to a double-blind, placebo-controlled study in which a 5-HT_3_ receptor antagonist attenuated rotavirus diarrhea in children (12), which confirmed a previous study with mice (13). Altogether, this suggests the participation of 5-HT, EC cells, and nerves in rotavirus illness, including secretory diarrhea (1, 2, 10, 11, 13, 14). It is hypothesized that rotavirus and/or NSP4 stimulates release of 5-HT by EC cells and subsequent stimulation of enteric nerves followed by chloride and water secretion from crypt cells (1, 10, 11).

The intestinal epithelium plays a key role in host defense mechanisms by maintaining a barrier against pathogens and toxic products in the lumen, with the intestinal tight junctions serving as the main regulator of the barrier function. Loss of tight junction integrity consequently opens the paracellular space between gut epithelial cells, facilitating the entry of harmful pathogens into the mucosa and resulting in inflammatory disorders and tissue injury. Beneath the intestinal epithelial cells, the ENS comprises a complex network of enteric neurons and enteric glial cells (EGCs) that control several intestinal functions. The central nervous system communicates with the ENS through both afferent and efferent nerves (15). Therefore, stimulation of the vagus nerve may accordingly modulate the intestinal epithelial cells or EGCs (16); indeed, efferent vagal nerve stimulation enforces the gut barrier (17–19). EGCs are a unique class of peripheral glial cells that nourish neurons and maintain ENS homeostasis. In the gut, they interact with several nonneuronal cell types: endothelial cells, enteroendocrine cells, enterocytes, and immune cells as well as the microbiota. A growing number of studies support the idea that EGCs are essential for gut integrity (20) and important local regulators of diverse gut functions such as motility, mucosal secretion, and host defense (20, 21). The mucosal EGCs have been recognized as active players in barrier function by secreting factors crucial for epithelial cell differentiation, such as *S*-nitrosoglutathione (GSNO) and glial cell-derived neurotrophic factor (GDNF) (22).

In a mouse model of intestinal injury, stimulation of the vagus nerve activated EGCs, which subsequently prevented burn-induced intestinal permeability and attenuated histological gut injury (17). Cheadle and coworkers (23) have shown that vagal nerve stimulation increases EGC activation, which is associated with better gut barrier integrity (24), and that the EGC-derived GSNO prevented epithelial barrier failure. Moreover, the activation of ECGs by a cholinergic agonist improves the intestinal barrier function following injury (24). Furthermore, GSNO protects against Shigella flexneri invasion *in vivo* by reducing barrier susceptibility (25). These findings all suggest that both the vagus nerve and neurotrophic factors contribute to maintaining the gut barrier.

As intestinal permeability is partly regulated by the vagus nerve and neurotrophic factors (17, 18, 23–26), we hypothesized that the vagus nerve and/or neurotropic factors may contribute to protecting the intestinal epithelial barrier during rotavirus insult.

## RESULTS

### The vagus nerve does not contribute to the maintenance of intestinal integrity during rotavirus infection in mice.

**FIG 1:**
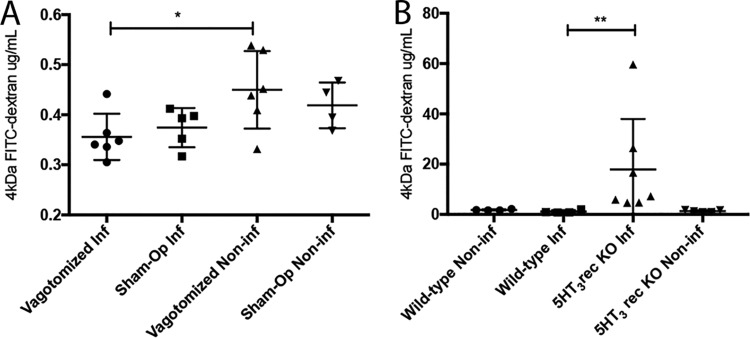
The vagus nerve does not affect the intestinal paracellular barrier in mice during rotavirus infection, but intrinsic signaling within ENS trough 5-HT_3_ receptors seems to be of importance. Subdiaphragmatic vagotomized and sham-operated adult BALB/c mice (A) and 5-HT_3_ receptor KO and wild-type C57BL/6 mouse pups (B) were orally infected with wild-type murine rotavirus (strain EDIM). At 45 h p.i., the mice received 10 μl of 4-kDa FITC-dextran orally. After 3 h, the blood was collected and passage of fluorescent dextran from intestine into the blood was measured with a fluorescence spectrofluorometer (494/518 nm). The fluorescence intensity values were correlated to a standard curve with known concentrations of 4-kDa FITC-dextran. Data are expressed as means with SD. (A) *t* test. *, *P < *0.05 (*n* = 4 to 6). (B) Mann-Whitney test. **, *P* < 0.005 (*n* = 4 to 6).

As vagus nerve stimulation can indirectly support gut barrier integrity during insult (19, 24, 27, 28), we investigated whether it plays a role in epithelial barrier homeostasis during rotavirus infection. Sham-operated and subdiaphragmatic vagotomized adult BALB/c mice were infected orally with murine rotavirus (strain EDIM) as described previously (7). At 45 h postinfection (p.i.), the mice received 4-kDa fluorescein isothiocyanate (FITC)-dextran orally (7) and were sacrificed 3 h later; the blood was collected and the fluorescence intensity in the serum was measured. The intestinal paracellular permeability of the FITC-dextran was decreased in infected vagotomized mice (*P < *0.05) compared to that in mock-infected vagotomized mice (Fig. 1A). Furthermore, no difference in permeability was observed between vagotomized and sham-operated infected mice, suggesting that the vagus nerve does not contribute to maintaining the gut barrier during rotavirus infection in mice (Fig. 1A).

### Serotonin signaling, through the 5-HT_3_ receptor, participates in regulation of intestinal permeability during rotavirus infection in mice.

To address the question of whether 5-HT and particularly the 5-HT_3_ receptor contribute to intestinal permeability, 5-HT_3_ receptor knockout (KO) mice were infected with rotavirus and permeability was investigated as previously described (7). We found that rotavirus-infected mice lacking the 5-HT_3_ receptor had significantly increased intestinal permeability (Fig. 1B).

### EGCs and nerves were in close proximity to EC cells and rotavirus-infected enterocytes in mouse small intestine.

**FIG 2:**
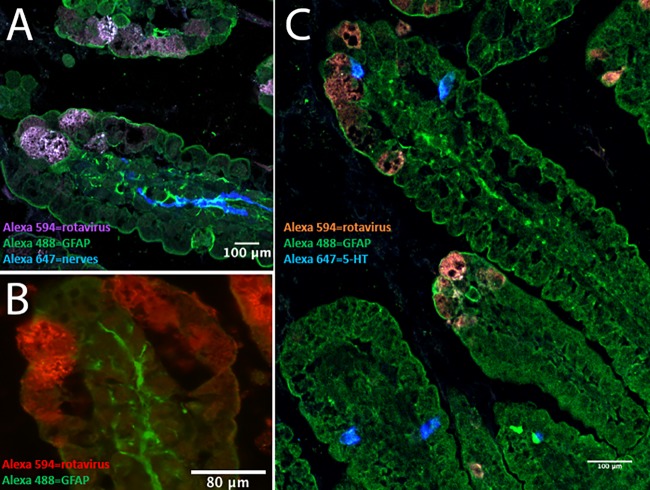
EGCs and nerves are in close proximity to EC cells and rotavirus-infected enterocytes in mouse small intestine. Infant BALB/c mice (5 to 7 days old) were infected for 24 h with 100 DD of murine rotavirus (strain EDIM), and the small intestine was processed for immunofluorescence. (A) Rotavirus-infected enterocytes (purple) in the duodenum are in close proximity to activated EGCs (green, GFAP staining), and activated EGCs are in proximity to the enteric nerves (blue). (B) Rotavirus-infected enterocytes (red) in the duodenum and activated EGCs (green, GFAP staining). (C) Rotavirus-infected cells (red), EC cells (blue, 5-HT staining), and activated EGCs (green, GFAP staining).

As EGCs are part of the ENS (20), which can be activated by rotavirus (10), we investigated the location of EGCs in relation to rotavirus-infected enterocytes and enteric nerves. Infant mice were infected with murine rotavirus strain EDIM (7) and sacrificed at 24 h p.i., and the small intestine was processed for immunohistochemistry. Figure 2 shows that the rotavirus-infected enterocytes appeared in proximity to EGCs, enteric nerves, and EC cells, which may facilitate cross talk.

### Serotonin and supernatant from rotavirus-infected human EC cells activated EGCs and induced GDNF release.

**FIG 3:**
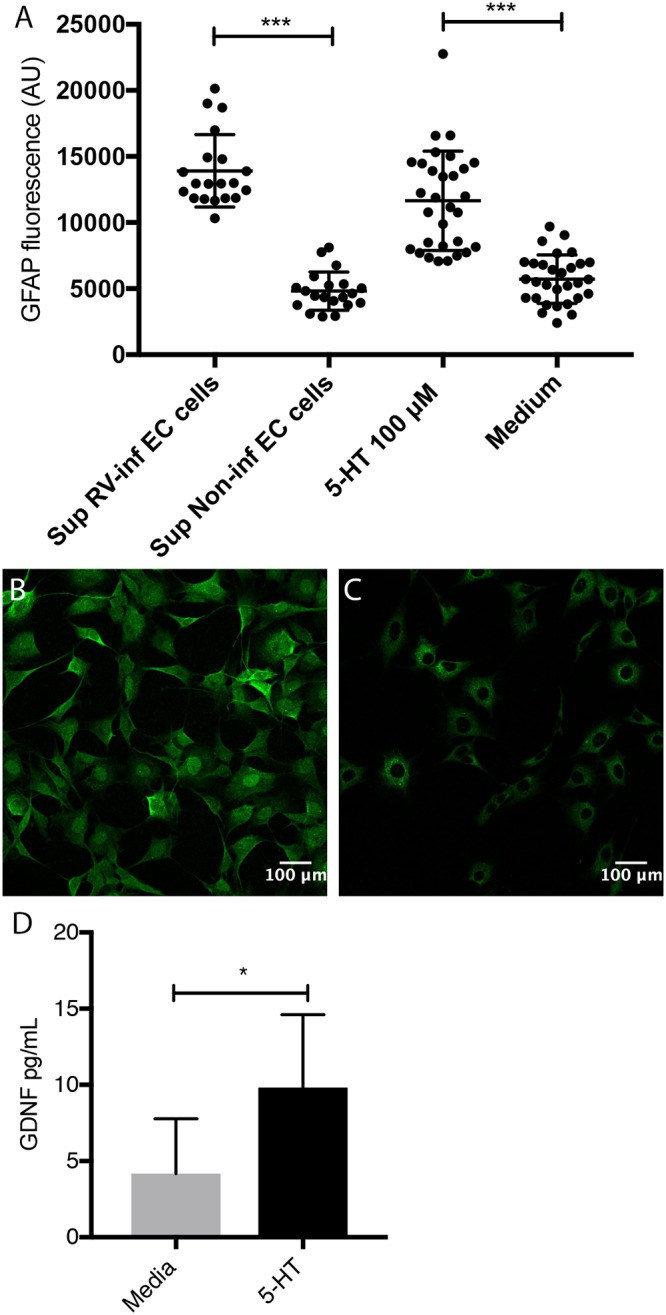
5-HT and supernatant from rotavirus-infected EC cells activate EGCs and induce GDNF release. (A) Supernatant from rotavirus-infected EC cells (MOI of 1; 24 h p.i.) (*n* = 20), but not noninfected cells (*n* = 20), activated the EGCs. EGCs were also activated following stimulation with 5-HT (100 μM) (*n* = 30), but not when exposed to only medium (*n* = 30). Activation was measured by quantification of fluorescence intensity of GFAP staining. (B and C) Fluorescence of GFAP staining in EGCs following stimulation by supernatant from rotavirus-infected (B) and noninfected (C) EC cells (green, Alexa Fluor 488). Images were acquired with a confocal microscope, and the average fluorescence intensity of single cell areas was measured using ImageJ. Data are presented as means and SD. ***, *P < *0.001, unpaired *t* test. (D) 5-HT induces GDNF release from EGCs *in vitro*. Rat EGCs, cultivated in a 24-well plate to confluence, were washed twice with cell medium and then stimulated with 5-HT (100 μM, dissolved in cell medium). GDNF release in the cell medium after 1-h stimulation was measured by ELISA. Control cells were exposed to the medium only. Data are means with SD. *, *P < *0.05, unpaired *t* test (*n* = 8).

The findings that EC cells respond with 5-HT release upon stimulation (29) and that EGCs express 5-HT receptors and are activated upon stimulation (30, 31) led us to ask whether supernatant from rotavirus-infected EC cells can activate EGCs. To address this question, EC cells were either infected (multiplicity of infection [MOI] = 1) with rhesus rotavirus (RRV) (11) or mock infected for 1 h, followed by washes and incubation with serum-free media. Cell supernatants were collected 24 h p.i., centrifuged at 580 × *g*, filtered through a 0.22-μm filter to remove cell debris, and then used to stimulate EGCs for 6 h. Only medium from infected EC cells, confirmed by enzyme-linked immunosorbent assay (ELISA) to contain 5-HT, led to increased EGC activation (*P < *0.001) (Fig. 3A). The EGCs were also activated by 5-HT alone (100 μM) (*P < *0.001) (Fig. 3A and B), as measured by quantifying the fluorescence intensity of the activation marker glial fibrillary acidic protein (GFAP) (32) (Fig. 3B and C). Next, we investigated if 5-HT could stimulate enterocytes to upregulate GDNF or if this is restricted to cross talk between EGCs and enterocytes. To address this question, we stimulated Caco-2 cells with 5-HT, 100 μM for 1 h, and stained for GDNF expression by immunofluorescence and GDNF release in supernatant and cell lysate by ELISA. Those experiments did not show any upregulation of GDNF by immunofluorescence (see Fig. S1 in the supplemental material), and GDNF protein in supernatant and cell lysate was under detection levels in the ELISA, suggesting that Caco-2 cells cannot respond with upregulation of GDNF following 5-HT stimulation.

10.1128/mBio.02834-19.1 FIG S1 5-HT does not affect epithelial Caco-2 cell GDNF expression. To investigate if enterocytes *in vitro* express and respond to increase in GDNF expression due to 5-HT stimulation, Caco-2 cells were cultured in monolayers and stimulated with 5-HT (100 μM) for 1 h. Immunofluorescence staining of GDNF was performed (green, Alexa Fluor 488). Stimulation with 5-HT (100 μM) did not affect the expression of GDNF within the cell (A) compared to that in unstimulated cells (B). The control consisted of Caco-2 cells stained without primary antibody (C). Download FIG S1, TIF file, 2.1 MB. Copyright © 2020 Hagbom et al. 2020 Hagbom et al. This content is distributed under the terms of the Creative Commons Attribution 4.0 International license.

An interesting question is whether factors other than 5-HT released from EC cells can activate EGCs. To address this question, supernatant from rotavirus-infected Caco-2 cells (24 h p.i.) (which do not produce 5-HT) were added to EGCs for 6 h of stimulation, followed by fixation and staining of the activation marker GFAP. No difference in GFAP activation could be observed between cells stimulated by supernatant from rotavirus-infected Caco-2 cells (24 h p.i.) and cells stimulated by supernatants from noninfected Caco-2 cells (24-h medium) (Fig. S2).

10.1128/mBio.02834-19.2 FIG S2 Supernatant from rotavirus-infected Caco-2 cells does not activate EGCs. EGCs were stimulated with supernatant from rotavirus-infected (24 h p.i.) and noninfected (24-h medium) Caco-2 cells for 1 and 6 h, respectively. Shown is fluorescence of GFAP staining in EGCs following stimulation by supernatant from noninfected (A) and rotavirus-infected (B) Caco-2 cells (green, Alexa Fluor 488). Activation was measured by quantification of fluorescence intensity of GFAP staining (C). Images were acquired with a confocal microscope, and the average fluorescence intensity of single cell areas was measured using ImageJ. Data are presented as arbitrary units (AU) and means with SD. There was no statistical difference using unpaired *t* test. Download FIG S2, TIF file, 2.1 MB. Copyright © 2020 Hagbom et al. 2020 Hagbom et al. This content is distributed under the terms of the Creative Commons Attribution 4.0 International license.

Next, we investigated whether EGC activation was associated with changes in intracellular calcium homeostasis upon stimulation by 5-HT. We used wide-field and confocal microscopy to investigate the fluorescence intensities of Fluo-4-labeled cells when exposed to both single and repetitive release of 5-HT from a microinjection capillary positioned near the cells. Exposure to a single release of 5-HT increased cytoplasmic Ca^2+^ in a proximity-related, wave-like manner, with cells closest to the capillary responding first and cells farther away responding later (Fig. S3). As the Ca^2+^ peak in the cells closest to the capillary subsided within minutes, the cytosolic Ca^2+^ in the cells farther away began to increase, meaning that the average cytosolic Ca^2+^ in the field of view remained at an increased level throughout the 10-minute experiment. Sequential addition of approximately 1 μl of 5-HT every 10 s from the capillary increased cytosolic Ca^2+^ in an accumulative manner (Fig. S3). The Ca^2+^ content of the cells therefore increased throughout the experiment. As new 5-HT was added repetitively, Ca^2+^ in the cells closest to the microinjection capillary continued to increase instead of subsiding, likely because there was no dilution of 5-HT due to diffusion.

10.1128/mBio.02834-19.3 FIG S3 5-HT affects cytosolic Ca^2+^ homeostasis in EGCs. EGCs were cultured in 35-mm coverslip-bottomed, poly-d-lysine-coated dishes and loaded with the Ca^2+^-responsive green fluorescent dye Fluo-4. Initially, 10 to 20 sequential images were captured at 10-s intervals to visualize a basal Fluo-4–Ca^2+^ average intensity. Subsequentially, 20 μl of 5-HT (100 μM) was loaded into a FemtoJet microinjector and released near the cells in focus. Sequential additions were made to investigate whether the cells would respond with increased Ca^2+^ in an accumulative, persistent manner. Approximately 1/20 of the needle content, i.e., ∼1 μl, was released at a time. The exposure time and number of Z-sections (3 or 4 sections) were kept to a minimum to reduce excessive photobleaching. Z-stacks were viewed as maximum intensity projections (MIPs). The images were analyzed using ImageJ. Download FIG S3, TIF file, 2.4 MB. Copyright © 2020 Hagbom et al. 2020 Hagbom et al. This content is distributed under the terms of the Creative Commons Attribution 4.0 International license.

We also investigated if 5-HT-activated EGCs could stimulate the release of GDNF. The EGCs were stimulated with 5-HT (100 μM, dissolved in medium) for 1 h; control cells were treated with medium. GDNF release into the medium was measured by ELISA). Figure 3D shows that 5-HT stimulated significant GDNF release (*P < *0.05) from the EGCs.

### GSNO increased the appearance of ZO-1 and maintained epithelial barrier integrity in rotavirus-infected polarized Caco-2 cell monolayers.

**FIG 4:**
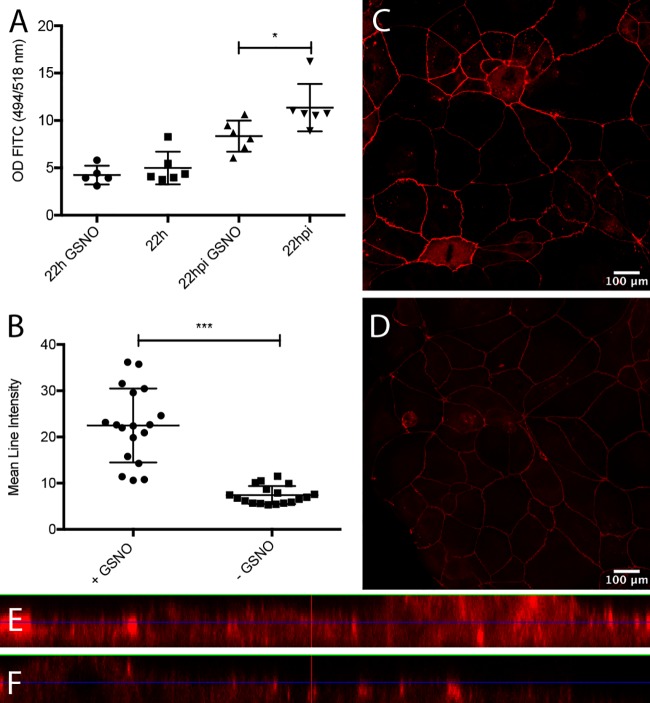
GSNO contributes to the maintenance of epithelial tightness of rotavirus-infected polarized Caco-2 cells and induces increased expression of ZO-1. (A) Polarized Caco-2 cells (>450 Ω/cm^2^) were stimulated with GSNO for 24 h and infected with rotavirus. At 6 h p.i., the apical medium was replaced with medium containing 4-kDa FITC-dextran (2 mg/Transwell insert), and samples were obtained from the basolateral side at 22 h p.i. to measure passage of FITC-dextran. The controls were noninfected cells with and without GSNO and infected cells without GSNO. Basolateral samples were analyzed for FITC-dextran by spectrophotometry (494/518 nm), and values are presented as the optical density (OD) values and means with SD. *, *P < *0.05, unpaired *t* test (*n* = 5 or 6). (B) GSNO increases ZO-1 tight junction protein in Caco-2 cell monolayers. Caco-2 cell monolayers cultured on Lab-Tek II chamber slides to near confluence with and without 24-h exposure to GSNO were stained for ZO-1 expression (red; Alexa Fluor 594). Confocal images were captured and the mean intensities on single-cell circumference were measured using ImageJ. (C and D) Monolayers of GSNO-treated (C) and untreated (D) Caco-2 cells. Data are mean fluorescence intensities and means with SD. ***, *P < *0.001, Mann-Whitney test (*n* = 17). (E and F) Deconvolved images visualized in ortho-mode with XZ and YZ intensities of GSNO-treated (E) and untreated (F) Caco-2 cells. The XZ and YZ selections in each image are positioned to cross the maximum number of high-intensity ZO-1-labeled cell borders.

As EGC activation helps to maintain gut barrier integrity (24) and GSNO prevents epithelial barrier failure (23) and restores the intestinal barrier after injury (24), including against S. flexneri invasion *in vivo* (25), we hypothesized that GSNO might protect against rotavirus insult. We cultivated Caco-2 cells in Transwell insert plates. After 4 to 5 days of cultivation, the Caco-2 cells were polarized (>450 Ω/cm^2^) and stimulated from the basolateral side with GSNO (80 μM) for 24 h following apical infection with rotavirus, essentially as described previously (33). GSNO significantly (*P < *0.05) reduced paracellular transport of 4-kDa fluorescein isothiocyanate (FITC)-dextran from the apical to basolateral domain in infected monolayers at 22 h p.i., but not in noninfected monolayers (Fig. 4A). Next, we investigated whether the reduced paracellular transport was associated with altered appearance of the tight junction-associated zona occludens 1 (ZO-1) protein. Figure 4B to F show that confluent Caco-2 cell monolayers stimulated with GSNO (80 μM) for 24 h had significantly higher ZO-1 fluorescence intensity than untreated cells (*P < *0.001), together suggesting that GSNO contributes to maintaining the intestinal epithelial barrier during rotavirus infection and increases the appearance of ZO-1 protein. Infection of Caco-2 cell monolayers without addition of extracullular GDNF did not increase ZO-1 expression, as determined by immunofluorescence (Fig. S4).

10.1128/mBio.02834-19.4 FIG S4 Rotavirus infection does not induce ZO-1 expression in epithelial cells. Caco-2 cells were infected with rotavirus for 6 h and stained for ZO-1 (red, Alexa Fluor 594) by immunofluorescence. Results for rotavirus infected (A) and noninfected (B) Caco-2 cell monolayers are shown. Download FIG S4, TIF file, 2.6 MB. Copyright © 2020 Hagbom et al. 2020 Hagbom et al. This content is distributed under the terms of the Creative Commons Attribution 4.0 International license.

### GSNO and GDNF improved the intestinal epithelial barrier *ex vivo* in mice and humans.

**FIG 5:**
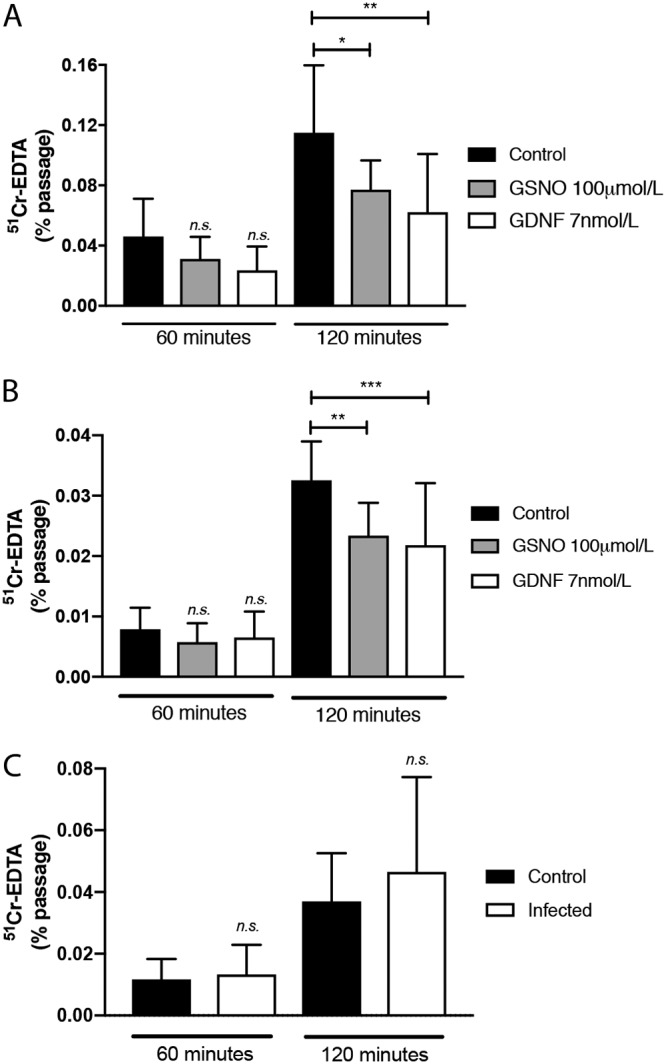
Effect of GSNO or GDNF on the paracellular permeability of mouse and human ileal resections. GSNO or GDNF decreased the passage of ^51^Cr-EDTA through a 4.9-mm^2^ ileal mucosal surface from either mice (*n* = 5 or 6) (A) or humans (*n* = 4) (B) over 120 min. (C) Infection did not alter the permeability of ileal resections from infected mice (*n* = 5 or 6 pooled replicates from two animals). Ussing chamber experiments were run for 120 min; samples (300 μl) were collected at 0, 60, and 120 min. Data are the means with SD from two-way ANOVA, followed by Tukey’s (A and B) or Bonferroni’s (C) multiple-comparison test. *, *P < *0.05; **, *P < *0.01; ***, *P < *0.001.

Ussing chamber experiments showed a stable transepithelial potential difference (PD) after equilibration in all tissues (Tables S1 to S3). The effect on the paracellular permeability of the neurotrophic factors GSNO and GDNF was measured *ex vivo* on ileal resections from humans and mice in an Ussing chamber setup. Both GSNO and GDNF significantly reduced the permeability in both the mouse (*P < *0.01) and human sections (*P < *0.001) (Fig. 5A and B). As rotavirus infection *in vivo* does not increase permeability (4–7), we were also interested in investigating if this effect could be confirmed *ex vivo*. This would suggest that our Ussing chamber results could be translated to an *in vivo* situation. The permeability in rotavirus-infected mice was unaffected and similar to uninfected control mice (Fig. 5C). None of the electrophysiological parameters were altered either by the treatments (Tables S1 and S2) or by rotavirus infection (Table S3).

10.1128/mBio.02834-19.7 TABLE S1 Effects of neurotrophic factors on electrophysiological parameters of human ileal mucosa mounted on Ussing chambers. Download Table S1, DOCX file, 0.01 MB. Copyright © 2020 Hagbom et al. 2020 Hagbom et al. This content is distributed under the terms of the Creative Commons Attribution 4.0 International license.

10.1128/mBio.02834-19.8 TABLE S2 Effects of neurotrophic factors on electrophysiological parameters of mouse ileal mucosa mounted on Ussing chambers. Download Table S2, DOCX file, 0.01 MB. Copyright © 2020 Hagbom et al. 2020 Hagbom et al. This content is distributed under the terms of the Creative Commons Attribution 4.0 International license.

10.1128/mBio.02834-19.9 TABLE S3 Electrophysiological parameters of rotavirus-infected ileal mucosa of mice, mounted on Ussing chambers. Download Table S3, DOCX file, 0.01 MB. Copyright © 2020 Hagbom et al. 2020 Hagbom et al. This content is distributed under the terms of the Creative Commons Attribution 4.0 International license.

### Rotavirus infection stimulated GDNF expression in bystander cells of mouse duodenum.

**FIG 6:**
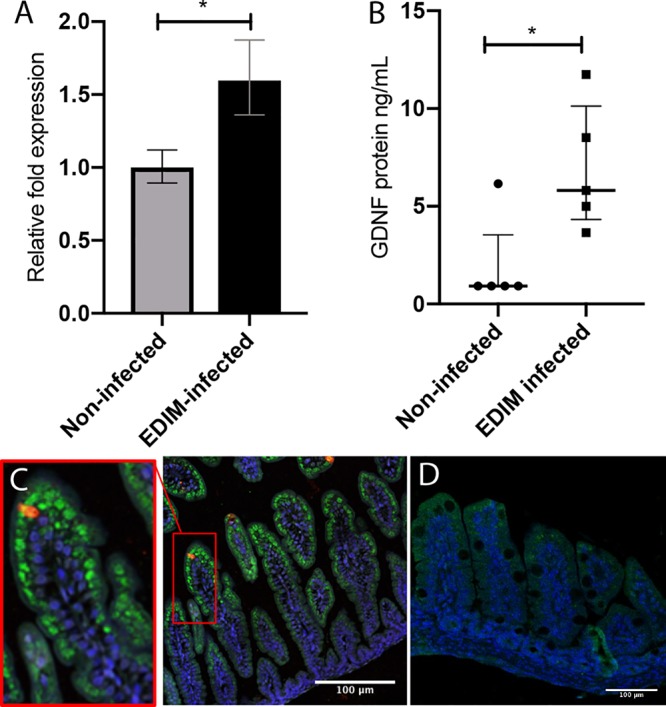
GDNF mRNA and protein levels were significantly higher in the duodenum of rotavirus-infected mice. (A) The duodenum of infected (*n* = 8) and noninfected (*n* = 7) mouse pups were collected at 16 h p.i., and the *Gdnf* and TATA-binding protein (*Tbp*) mRNA levels were quantified using qPCR. There was significant upregulation of *Gdnf* mRNA in the infected mice. Data are means ± SEM. *, *P < *0.05, unpaired *t* test. (B) The duodenum of infected and noninfected mouse pups were collected at 16 h p.i., and the GDNF protein levels were measured using ELISA. The infected mice had significantly higher levels of GDNF protein than the noninfected controls. Four biopsy specimens from noninfected pups had GDNF protein concentrations below the ELISA detection limit. Data are medians with interquartile range. *, *P < *0.05, Mann-Whitney test (*n* = 5). (C and D) GDNF was present in the enterocytes of mouse small intestine, mainly in the middle and tip of the villi. Immunofluorescence staining shows GDNF (green, Alexa Fluor 488) in the ileal enterocytes of rotavirus-infected mouse pups (red, Alexa Fluor 594) at 16 h p.i. (C) and noninfected mouse pups (D). The tissue was counterstained with 4′,6‐diamidino‐2‐phenylindole (DAPI) nuclear stain.

GDNF is critically involved in intestinal epithelial wound healing and the direct promotion of barrier maturation and enterocyte proliferation (22). As rotavirus causes significant lesions in the small intestine but does not impair the intestinal barrier (7), and GSNO contributes to maintaining the epithelial barrier during rotavirus infection in Caco-2 cells (Fig. 4), we next asked whether rotavirus infection could stimulate GDNF production *in vivo*. To answer this question, we mock infected infant mice or infected them with rotavirus. At 16 h p.i., the mice were sacrificed and the duodenum processed for the GDNF mRNA and protein contents. Figure 6A and B show that rotavirus infection resulted in significantly increased expression of GDNF mRNA (average, 1.6-fold; *P < *0.05) (Fig. 6A) and protein (*P < *0.05) (Fig. 6B) in the duodenum compared to that in uninfected mice, suggesting that a viral infection of the gut can stimulate the expression and release of a neurotrophic factor associated with wound healing and barrier maturation, as well as enterocyte proliferation. Apart from that, GDNF expression was higher in infected tissue than in uninfected tissue; expression was strongest in the middle and top of the villi rather than the crypts (Fig. 6C). Most interesting was the observation that uninfected bystander cells, probably enterocytes (based on the number of cells), of infected animals had significant higher expression of GDNF than did uninfected mice (Fig. 6C and D). To address the question of whether GDNF is expressed by enterocytes without any stimulation (without signals from EGCs or EC cells or virus), Caco-2 cells were grown as monolayers and stained for GDNF, both uninfected cells and cells 6 h postinfection. As shown in Fig. S1, GDNF was expressed in unstimulated Caco-2 cells but was not increased by rotavirus infection *in vitro* (Fig. S5).

10.1128/mBio.02834-19.5 FIG S5 Rotavirus infection of enterocytes *in vitro* did not induce increase of GDNF expression. Monolayers of Caco-2 cells were infected with rotavirus (MOI = 1) and stained for GDNF expression 6 h p.i. No difference in GDNF fluorescence (green, Alexa Fluor 488) was observed in infected (A) and noninfected (B) Caco-2 cells, as determined by immunofluorescence staining. The control consisted of Caco-2 cells stained without primary antibody (C). Download FIG S5, TIF file, 2.8 MB. Copyright © 2020 Hagbom et al. 2020 Hagbom et al. This content is distributed under the terms of the Creative Commons Attribution 4.0 International license.

Next, we investigated if the lack of effect on permeability by vagotomy (Fig. 1) was due to alterations of GDNF concentration in the gut tissue. To address this question, duodenal tissues from sham-operated and vagotomized infected mice were extracted and examined for GDNF by ELISA. Vagotomy did not affect the concentration of GDNF in duodenal tissues (Fig. S6).

10.1128/mBio.02834-19.6 FIG S6 Vagotomy did not affect the concentration of GDNF in mice duodenal tissues. Duodenal tissues from sham-operated and vagotomized infected mice were extracted and examined for GDNF by ELISA. There was no significant difference of GDNF levels in duodenums of rotavirus-infected vagotomized and rotavirus-infected sham-operated mice. Data are means with SD; *P* = 0.9372, Mann-Whitney test (*n* = 6). Download FIG S6, TIF file, 0.9 MB. Copyright © 2020 Hagbom et al. 2020 Hagbom et al. This content is distributed under the terms of the Creative Commons Attribution 4.0 International license.

## DISCUSSION

In the present study, we investigated the potential mechanisms that maintain the gut barrier during rotavirus insult. The rationale was that previous studies have reported that rotavirus leaves the intestinal permeability unaffected or reduced during diarrhea in humans (4–6) and mice (7). Using subdiaphragmatic vagotomized mice, we found that the vagus nerve, at least at the level of diaphragmatic vagotomy, did not contribute to protecting the paracellular barrier during infection (Fig. 1A). The absence of participation by the vagus nerve in our experimental setup might be partly related to the modest inflammatory response, a hallmark of rotavirus infection (10, 34–37). Supporting this is the finding that vagal nerve stimulation can protect against burn-induced inflammatory intestinal injury (17, 19). Under homeostasis conditions, the epithelial surfaces form a highly selective permeability barrier that prevents the passage of toxic proinflammatory molecules from the external milieu into the submucosa and for the systemic circulation. The loss of this barrier integrity could allow transmucosal access to normally excluded luminal substances, e.g., endotoxin and microbes, which may lead to inflammation and tissue injury (38–40). While rotavirus does not affect the gut paracellular barrier *in vivo*, viremia and extramucosal spread have been documented (1). How the virus or antigen can disseminate from the gut without affecting the gut barrier remains unresolved but may include sampling by dendritic or M cells.

EGCs may affect local regulation of the epithelial barrier through the close interaction between intestinal epithelial cells, EC cells, and nerves. EGCs are located not only in the myenteric plexa of the ENS but also in the submucosa with projections toward the basolateral side of epithelial cells, which can be activated and release neurotropic factors that directly affect epithelial permeability, possibly via tight junction proteins (41). More interestingly, we observed higher GDNF expression in enterocytes at the middle and top of the villi than in the crypts and higher expression in uninfected bystander cells of infected animals than in uninfected animals (Fig. 6C and D). Based on these observations, we speculate that GDNF in enterocytes affects tight junction protein expression in an autocrine manner and also acts as a paracrine communicator between infected and uninfected bystander cells. Indeed, immunofluorescence staining of enterocytes *in vitro* showed GDNF expression (Fig. S1), and it has also previously been shown that GDNF is expressed on enterocytes (22) and that enterocytes have receptors for GDNF and can bind GDNF from EGCs. Alternatively, the close positioning of rotavirus-infected enterocytes, EGCs, EC cells, and nerves in the gut facilitates cross talk signaling. Supporting this is the fact that GDNF is secreted from the ENS containing glial cells (22, 42). Moreover, Gabella (43) found synapse-like junctions between enteric neurons and EGCs, suggesting communication between neurons and EGCs in the ENS, and Bohorquez et al. (44) found that EGCs cross talk with enteroendocrine cells. *In vitro*, we did not observe increased GDNF expression in Caco-2 enterocytes during rotavirus infection (Fig. S5), which may indicate the need for interaction with EGCs and glial cell-derived GDNF. Thus, based on previous observations and those made in this work, the increased expression of GDNF on enterocytes *in vivo* is probably a combined autocrine/paracrine effect between infected and bystander cells and cross talk between infected enterocytes, EGCs, EC cells, and nerves.

5-HT and the supernatant from infected EC cells increased the glial cell activation marker GFAP, and 5-HT increased cytosolic calcium in cultured EGCs (Fig. S3). While 5-HT is a major neurotransmitter released from EC cells, we cannot prove *per se* that it was the only contributing factor in the medium that activated the EGCs. However, the facts that EC cells respond with 5-HT release upon rotavirus stimulation (11) and that EGCs express 5-HT receptors and become activated upon 5-HT stimulation (31) support the premise that rotavirus-infected/stimulated EC cells activate EGCs. In addition, we found that the neurotrophic factor GDNF was released from EGCs following 5-HT stimulation (Fig. 3D), which is of interest, as GDNF has gut barrier protective properties (22). The fact that 5-HT_3_ receptor KO infected mice demonstrated increased intestinal permeability (Fig. 1B) compared to that of wild-type mice further supports the hypothesis that 5-HT and particularly 5-HT_3_ receptors contribute to maintain the intestinal barrier during rotavirus infection. Since the vagus nerve and thus vagal 5-HT_3_ receptors do not play a role in the maintained intestinal epithelial barrier during rotavirus infection, it is most probably an intrinsic regulation within the ENS.

EGCs and the released GSNO can protect the intestinal barrier and increase the expression of tight junction proteins during inflammation and inflammatory bacterial infection (23, 25, 45). In accordance with these observations, we found that GSNO protected the paracellular barrier of infected polarized Caco-2 cells, as assessed with the transmural passage of FITC-dextran (7, 46). The finding that permeability was increased in untreated infected Caco-2 cells is in contrast to the case with infection *in vivo*, probably due to the absence of nerves and neurotrophic factor-producing glial cells *in vitro*. We do not consider increased intestinal permeability a major contributing factor of rotavirus diarrhea; rather, we have found in this work and elsewhere (4–7) that the epithelial barrier becomes tighter or remains unaffected during rotavirus diarrhea, suggesting that rotavirus diarrhea is secretory, driven by active chloride ion secretion from the crypt cells (10).

We used Ussing chambers to investigate whether neurotrophic factors could influence barrier function *ex vivo* in mouse and human ileal resections. A major finding was that both GSNO and GDNF enhance barrier function, as measured by decreased passage of ^51^Cr-EDTA (Fig. 5). We also found that the small intestines of rotavirus-infected mice showed no significant changes in permeability compared to uninfected tissue, confirming a previous observation in mice (7). This may be due to the infection-increased expression of GDNF (Fig. 6). GSNO prevents S. flexneri-induced barrier lesions in ileal loops *in vivo*, and EGCs significantly reduce barrier lesions and the inflammatory response induced by S. flexneri in Caco-2 cell monolayers (25). This suggests that the barrier-protective effects of GSNO and GDNF are operational in both inflammatory (S. flexneri) and noninflammatory (rotavirus) microbial insults. While the protection mechanism of barrier function is unresolved, Meir et al. (42) have shown that both the Caco-2 and HT29/B6 epithelial cell lines express the GDNF receptors RET, GFRα1 (GDNF family receptor alpha 1), and GFRα2. The authors proposed that GDNF inhibits the p38-MAPK (mitogen-activated protein kinase) pathway as a mechanism to protect barrier function.

**FIG 7:**
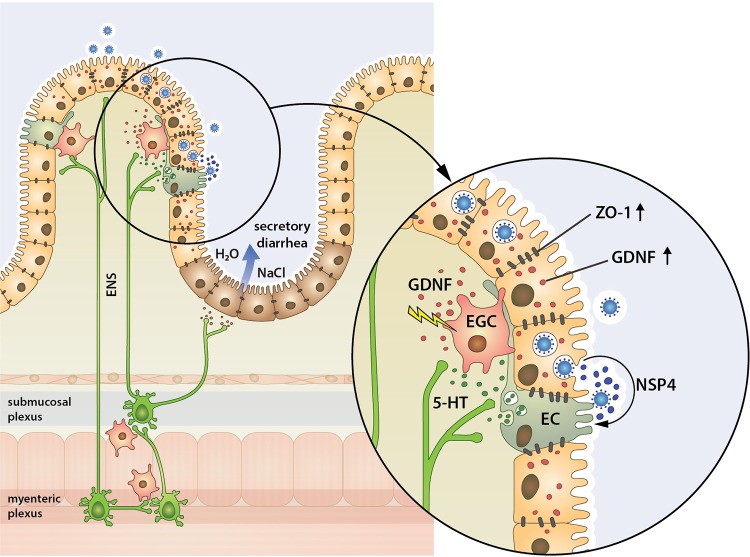
Proposed mechanism for how neurotrophic factors protect the intestinal barrier from rotavirus insult. Studies with humans and mice (4–7) have shown that the noninflammatory infection with rotavirus leaves the intestinal permeability unaffected or even reduced during diarrhea, in contrast to most bacterial infections (8, 9). Gastrointestinal permeability is regulated by the vagus nerve and the enteric nervous system (ENS), which is composed of neurons and EGCs. Rotavirus infects mature enterocytes on the top and middle of villi, which results in release of virus and at least the enterotoxin NSP4, which stimulate EC cells. 5-HT is contained in secretory granules of the EC cells and is released following stimulation by rotavirus and NSP4 (11). Released 5-HT activates EGCs to increase release of GDNF, which subsequently increase the tight junction protein ZO-1 in infected and bystander cells. It may also be that infected enterocytes in a paracrine manner stimulate increase of GDNF in bystander cells. Intrinsic enteric 5-HT_3_ receptors are of importance in the regulation of barrier function during rotavirus insult, since mice lacking this receptor have an increased permeability during rotavirus infection. The proposed mechanism is based on *in vitro* and *ex vivo* studies and is modified from previous disease models (1–3).

In conclusion, our results provide for the first time a possible mechanism for how the gut barrier can remain unaffected during rotavirus infection in human and mice (4–7). We showed by *in vitro*, *ex vivo*, and *in vivo* experiments that the neurotrophic factors GSNO and GDNF from EGCs and enterocytes both have intrinsic properties to protect the gut barrier from rotavirus insult. We also showed that enteric intrinsic 5-HT_3_ receptors are of importance for the epithelial barrier, whereas extrinsic signaling through the vagus nerve does not play a role in intestinal barrier during rotavirus infection. The observations advance our understanding of how the gut barrier can respond to the noninflammatory rotavirus insult and bring new information into the model of secretory rotavirus diarrhea and gut homeostasis (Fig. 7).

## MATERIALS AND METHODS

### Cells.

Cells of the human epithelial cell line Caco-2 (ATCC HTB-37), established from a colon adenocarcinoma with intestinal origin, were employed for *in vitro* experiment for epithelial tightness. Rat EGCs (ATCC CRL-2690) and the human enterochromaffin cell line GOT1 (47) were used for investigating responses to rotavirus. EGCs were grown in Dulbecco’s modified Eagle’s minimum essential medium (DMEM; Thermo Fisher Scientific [Sweden], GIBCO; code 11995-065) with high glucose (4.5 mg/ml), 1 mM sodium pyruvate, and 4 mM l-glutamine and supplemented with 10% fetal bovine serum and gentamicin (100 μg/ml). GOT1 cells were grown in RPMI medium (Fisher Scientific, Lonza; code BE12-167F), supplemented with 10% fetal bovine serum, 1 mM sodium pyruvate, 1× minimal essential medium (MEM) nonessential amino acids (GIBCO; code 11140050), 5 mM l-glutamine, and gentamicin (100 μg/ml). Cells were cultivated at 37°C in an atmosphere of 5% CO_2_ and 95% humidity. For *in vitro* infection studies, rhesus rotavirus was used and infection performed as previously described (48).

### Animals.

Rotavirus-naive BALB/c adult (8 weeks old) and infant (5 to 7 days old) mice were purchased from B&K Laboratories, Sollentuna, Sweden, and were used for glial cell assessment in the small intestine. Subdiaphragmatic bidirectional vagotomized and sham-operated adult BALB/c mice and C57BL6J mice and wild-type and serotonin receptor-3A KO mice (B6.129 × 1-Htr3^atm1Jul^/J) were purchased from The Jackson Laboratory (Sacramento, CA) and Envigo (the Netherlands) and used for permeability studies.

Mice were used and housed in standard cages with free access to food and water. Pregnant females were transferred to individual cages 1 week before the expected day of birth, and offspring remained with their mothers during the experimental period.

Mice were orally infected with 10 μl/animal (100 DD_50_ diarrhea doses) of wild-type murine rotavirus (strain EDIM) as previously described (7, 11). All procedures were performed according to ethical approvals N498/11 and N276/12 by the ethical committee in Linköping, Sweden.

### Human biopsy specimens.

Macro- and microscopically normal specimens from the neoterminal ileum, or terminal ileum next to the ileocecal valve, were obtained during surgery for colonic cancer from 7 patients (3 men) aged 77.4 ± 7.8 years (mean ± standard deviation [SD]) at the University Hospital of Linköping. The patients had no generalized disease, and none had received preoperative chemo- or radiotherapy. The Regional Ethical Review Board, Sweden, approved the study, and all subjects gave their written informed consent.

### Antibodies.

The following primary antibodies were used: guinea pig anti-rotavirus (1/200, in-house sera), rabbit anti-GFAP (1/200; Dako Cytomation; code Z0334), mouse anti-PGP.9.5 (1/200; Thermo Fisher, Invitrogen; code MA1-83428), mouse anti-5-HT (1/200; Dako Cytomation; code M0758), rabbit anti-GDNF (1/200; Invitrogen; code PA118359), and rabbit anti-ZO-1 (1/100; Zymed; code 40-2300). As secondary antibodies, the following were used: goat anti-rabbit Alexa Fluor 488/594 (1/200; code 111-545-144/111-585-144), goat anti-mouse Alexa Fluor 488/594/647 (1/200; code 115-545-003/115-585-166/115-605-146), and donkey anti-guinea pig Alexa Fluor 594 (1/200; code 106-585-003). Nuclear staining was done with 5 μg/ml of 4′,6-diamidino-2-phenylindole (DAPI; Invitrogen; code D1301). Mounting media used were from Dako Cytomation (code S3023).

### *In vitro* permeability assay.

An *in vitro* permeability assay was performed to investigate whether GSNO had any effect on epithelial monolayer barrier function during rotavirus infection. Caco-2 cells at a density of 400,000/well were seeded in Transwell inserts of 12-well plates (pore size, 0.4 μm; Costar; 3460). After 4 to 5 days of cultivation, transepithelial resistance was measured to confirm that cells had been polarized and established expected barrier characteristics (>450 Ω/cm^2^). Cells were then stimulated with GSNO for 24 h, where 20 μl was added to the basolateral side to a final concentration of 80 μM. Medium was replaced with serum-free medium, and cells were infected with rotavirus at a multiplicity of infection (MOI) of 1 essentially as described previously (33). After 1 h of infection, virus was washed away and fresh serum-free medium (DMEM supplemented with l-glutamine and gentamicin) added to each insert with cells. GSNO was added basolaterall (80 μM). At 6 h postinfection (h p.i.), apical medium was replaced with medium containing 4-kDa FITC-dextran, 2 mg/Transwell insert (Sigma-Aldrich; code FD4), and 100-μl samples were taken from the basolateral side at 22 h p.i. Samples were diluted 1:50 in Milli-Q water and analyzed for FITC (494/518) in a spectrofluorometer (Cary Eclipse; Varian, Australia). Samples containing known concentrations of FITC-dextran were measured for the standard curve.

### *In vivo* intestinal permeability assay.

Permeability determination *in vivo* was performed to investigate the role of the vagus nerve and the 5-HT_3_ receptor in the regulation of intestinal epithelial barrier function during rotavirus infection. Subdiaphragmatically vagotomized and sham-operated adult BALB/c mice and 5-HT_3_ receptor KO and wild-type C57BL/6 mouse pups were infected with 100 diarrhea doses (100 DD_50_ diarrhea doses) of wild-type murine rotavirus strain EDIM. At 45 h p.i. mice were given 4-kDa FITC-dextran (2.5 mg/kg of body weight for adult mice and 0.7 mg/infant mouse) (Sigma-Aldrich; catalog no. FD-4), as previously described (7). At 48 h p.i., blood was collected in serum tubes (BD Microtainer serum tubes; catalog no. 365968), set to clot, and centrifuged for 5 min at 2,500 × *g*. Serum was subsequently diluted 1/50 in Milli-Q water, and fluorescence intensities of FITC (494/518 nm) were measured with a fluorescence spectrofluorometer (Cary Eclipse; Varian, Australia). The fluorescence value was correlated to a standard curve, obtained from values of samples with known concentrations (in micrograms per milliliter) of 4-kDa FITC-dextran.

### Immunofluorescence staining of ZO-1.

Caco-2 cells grown on Lab-Tek II chamber slides were stimulated with GSNO (80 μM, diluted in cell medium) for 24 h and then stained for zonula occuldens (ZO-1). Caco-2 cells were also infected with RRV (MOI = 1) for 6 h. Cells were fixed for 10 min with 4% paraformaldehyde (PFA), washed with phosphate-buffered slaine (PBS), and permeabilized with 0.2% Triton-X for 10 min. After washing, blocking was performed with 1% bovine serum albumin (BSA) in PBS for 60 min, followed by primary antibody incubation for 60 min (1:100, rabbit anti-ZO-1), 3 washings with PBS, and incubation with secondary antibody for 60 min (goat anti-rabbit Alexa Fluor 594). Slides were washed 4 times with PBS, and coverslips were mounted with fluorescence mounting medium. Pictures were captured using confocal microscopy (LSM700; Zeiss).

### Immunofluorescence staining of EGCs, nerves, EC cells, and GDNF in intestinal segments.

Paraffin-embedded intestinal biopsy specimens from the duodenum, jejunum, and ileum were cut in 5-μm sections and dried on glass slides at 60°C for 2 h. Deparaffination was performed with Aqua de Par (Histolab Products AB, Gothenburg, Sweden; code BC-ADP1002M) for 10 min at 80°C. Antigen retrieval was performed in a retriever cooker (2100 retriever; Histolab) with rodent decloaker retrieval buffer (Histolab; code BC-RD913M) and was ended when a temperature of 121°C was reached. Slides were transferred to Tris-buffered saline (TBS; Histolab; code BC-TWB946L2J) for 10 min, blocked with rodent block solution (Histolab; code BC-RBM961H) for 15 min, washed with TBS, and incubated with primary antibody mix (1:200 in TBS) of rabbit anti-GFAP, rabbit anti-GDNF, guinea pig anti-rotavirus, and mouse anti-5-HT or anti-PGP.9.5 for 90 min at room temperature (RT). Slides were washed 3 times with TBS, and a mixture of secondary antibodies (1:200 in TBS; goat anti-rabbit Alexa Fluor 488, goat anti-guinea pig Alexa Fluor 594, and goat anti-mouse Alexa Fluor 647) was added to the tissue and incubated for 60 min at RT in the dark. Slides were washed 4 times with TBS, and coverslides were mounted with fluorescence mounting medium. Pictures were captured using confocal microscopy (LSM700; Zeiss, Oberkochen, Germany). ImageJ software was used to measure the mean intensities on single-cell circumference.

### EGC stimulation *in vitro*.

EGCs were stimulated with supernatant from rotavirus-infected enterochromaffin (EC) cells, supernatant from rotavirus-infected Caco-2 cells, and 5-HT. EC cells (GOT1) and Caco-2 cells were grown in 6-well plates and infected with rotavirus as previously described (33). Noninfected cells were used as a negative control. Cell supernatants were collected 24 h p.i., centrifuged at 580 × *g*, filtered through a 0.22-μm filter to remove cell debris, and then used to stimulate EGCs. Rat EGCs were grown on Lab-Tek II chamber slides and stimulated with the cell supernatants and 5-HT (100 μM, dissolved in cell medium). Supernatants from noninfected EC and Caco-2 cells and only medium served as controls. Stimulation was performed at 37°C in 5% CO_2_. Six-hour stimulation was performed for investigation of GFAP expression by immunofluorescence.

To investigate release of GDNF, EGCs were cultivated in a 24-well plate to confluence. Cells were washed twice with fresh cell medium before supernatant from rotavirus-infected Caco-2 cells (24 h p.i.) or 5-HT-containing medium (100 μM) was added for 1 h of stimulation. Control cells were washed twice and received supernatant from noninfected Caco-2 cells (24-h medium) and fresh media. Stimulation was performed at 37°C in 5% CO_2_.

### Determination of serotonin and GDNF.

Determination of serotonin and GDNF was performed by ELISA as described by the manufacturer (IBL International, Hamburg, Germany [code RE59121], or Nordic Bio Site AB, Sweden [codes EKR50 and EKM176]).

### Immunofluorescence staining of GFAP and GDNF.

Rat EGCs and Caco-2 cells on Lab-Tek II chamber slides were fixed with ice-cold acetone for 10 min and 4% formaldehyde for 30 min, respectively. Formaldehyde-fixed cells were treated with 0.2% Triton X-100 for 10 min and washed twice with PBS.

Briefly, specimens were washed with PBS and blocked with 5% BSA in PBS for 60 min at RT. Primary antibody, rabbit anti-GFAP or rabbit anti-GDNF, was added to the cells and incubated for 1 h at RT. Following 3 washes with PBS, secondary goat anti-rabbit IgG Alexa Fluor 488 was added and incubated for 1 h at RT. Specimens were washed 3 times with PBS and mounted with fluorescence mounting medium, and fluorescence was examined by confocal microscope (LSM700; Zeiss). For quatification, ImageJ software was used to measure the mean intensities on single-cell areas.

### Ussing chamber experiments.

We employed Ussing chambers in experiments to investigate the potential role of EGC products in controlling ileal mucosal permeability. Ileal segments from 5 or 6 mice and healthy ileum from 7 colon cancer patients were directly after dissection put in oxygenated Krebs buffer and transported to the laboratory. Segments of villus epithelium (VE) was identified and dissected from mouse and human tissue and mounted in Ussing chambers (Harvard Apparatus Inc., Holliston, MA) as previously described (49, 50). Mucosal compartments were filled with 1.5 ml of cold 10 mM mannitol in Krebs buffer, and the serosal compartments were filled with 1.5 ml of 10 mM glucose in Krebs buffer. The exposed surface area between the mucosal and serosal sides was set at 4.9 mm^2^. After mounting of tissue, the chambers were kept at 37°C and continuously oxygenated in 95% O_2_–5% CO_2_ and circulated by gas flow. Before the experiments were started, tissues were equilibrated for 30 min in the chambers to achieve steady-state conditions in transepithelial potential difference (PD), with two replacements of 37°C mannitol or glucose buffer at 10 and 20 min. The short-circuit current (Isc) and transepithelial resistance (TER) and PD were monitored throughout the experiments. For more details on Ussing chamber experiments, see reference 50.

### Paracellular permeability.

Ileal VE segments were mounted in triplicates in Ussing chambers. ^51^Cr-EDTA (molecular weight [MW], 384 Da; Perkin-Elmer, Boston, MA) was used as a paracellular probe, added to the mucosal side to a final concentration of 34 μCi/ml. To investigate whether EGC-derived neurotropic factors influence the control of intestinal permeability, GSNO (100 μmol/liter) (45) or GDNF (7 nmol/liter) (42) was added to the serosal side just after collecting the first serosal sample at time zero. Serosal samples (300 μl) were further collected at 60 and 120 min after the start. Samples were saved for measuring ^51^Cr-EDTA permeability as described below. Permeability was calculated during the 60- to 120-min period. Collection tubes were placed in a gamma counter (1282 Compugamma; LKB, Bromma, Sweden) for ^51^Cr-EDTA measurements. ^51^Cr-EDTA permeability was given as percent passage.

### Calcium imaging.

EGCs were cultured in 35-mm-coverslip-bottomed, poly-d-lysine coated MatTek microwell dishes (MatTek Corporation, Ashland, MA; code P35GC-1.5-10-C) and loaded with the Ca^2+^-responsive green fluorescent dye Fluo-4 (Fluo-4 NW calcium assay kit; Molecular Probes, Eugene, OR), dissolved according to the manufacturer’s protocol. For each 35-mm dish, 1 ml was used, and cells were incubated at 37°C with the dye for 30 to 45 min before microscopy.

Initially, 10 to 20 sequential images were captured with 10-s intervals to visualize a basal Fluo-4-Ca^2+^ average intensity. Subsequently, 20 μl of 5-HT (100 μM) was loaded into a FemtoJet (Eppendorf) microinjection, from which 5-HT was released in the near vicinity of the cells in focus. Using the microinjection capillary, sequential additions were also performed to investigate whether cells would respond with Ca^2+^ increases in an accumulative, persistent manner. Approximately 1/20 of the needle content, i.e., 1 μl, was released at a time. Exposure time and number of Z-sections (3 or 4 sections) were kept at a minimum to reduce excessive photobleaching. Z-stacks were viewed as maximum intensity projections (MIPs). The aperture correlation confocal module and the peripherals associated with the microscope were controlled via Zeiss Zen software. Image analysis was done using ImageJ (51). Wide-field and confocal time-lapse microscopy was carried out using a VivaTome (Zeiss, Oberkochen, Germany) module mounted on an inverted Zeiss Axio Observer.Z1. Illumination in the VivaTome was obtained using a metal halide (HXP 120C) illumination source. A 12-bit AxioCam MRm (Zeiss) charged-coupled device (CCD) was used to acquire images of wide-field fluorescence and VivaTome confocal, side by side on the split camera chip. Images were captured using an LD Plan-Neofluar 20×/0.4 or a Plan-Apochromat 40×/1.4 objective.

### Protein extraction and measurement of GDNF protein concentration.

Extraction of proteins was performed on segments of duodenum collected from each mouse at 16 h p.i. T-per tissue protein extraction reagent (Thermo Fisher Scientific, Waltham, MA) was used following the manufacturer’s instructions.

The concentration of GDNF was measured in the protein lysate using a commercial ELISA kit (Nordic BioSite AB, Täby, Sweden; code EKM176) following the manufacturer’s instructions. The measured protein concentrations were divided by the weight of the respective duodenum biopsy specimen (ranges, 15.3 to 22.0 mg and 16.1 to 25.1 mg for infected and noninfected samples, respectively). A value corresponding to half the detection limit of the ELISA was given for samples with protein levels below the detection limit of GDNF.

### Extraction of RNA, reverse transcription, and quantitative PCR for *GDNF* mRNA from the duodenum.

Segments from the duodenum were collected from each mouse at 16 h p.i. The RNA was extracted using an RNeasy Plus universal minikit (Qiagen, Hilden, Germany) following the provided instructions. To confirm the absence of DNA, quantitative PCR (qPCR) specific for the TATA-binding protein gene (*TBP*) was performed on the RNA extract. The concentration of RNA was measured with a NanoDrop ND-1000 spectrophotometer (Saveen Werner, Life Science, Sweden). Following this, reverse transcription using 1 μg of RNA was carried out with an iScript cDNA synthesis kit (Bio-Rad, Uppsala, Sweden) following the manufacturer’s instructions. cDNA was quantified with SYBR green-based quantitative PCR with *TBP* as the reference gene. PrimePCR primers for *GDNF* and *TBP* (Bio-Rad) were used. The qPCR was performed with a CFX96 real-time system (Bio-Rad) with the following conditions: first, a denaturation was performed for 2 min at 95°C, followed by 45 cycles of 5 s at 95°C and 30 s at 60°C and a melting-curve analysis.

Negative controls for cDNA synthesis, RNA extraction, and no template control (NTC) were included. Results were exported from CFX Maestro software and analyzed using the ΔΔ*C_T_* method and are presented as relative fold expression.

### Statistics.

Statistical analysis was performed with GraphPad Prism 8.0 for Mac 1.0.

Continuous variables are presented as mean with standard deviations, and unpaired *t* test was used to test differences between two groups if the variables followed normal distribution as determined by the Shapiro-Wilks test. Otherwise, the variables were presented as median with interquartile range, and Mann-Whitney U test was used to test differences between two groups.

Ussing chamber data were analyzed by one-way analysis of variance (ANOVA) followed by Tukey’s test (noninfected versus infected mice) or by two-way ANOVA followed by Dunnett’s test (mice and humans treated with GSNO or GDNF).
