# Supplementary material for: Neurotrophic Factors Protect the Intestinal Barrier from Rotavirus Insult in Mice
Source: mBio. 2020 Jan 21;11(1):e02834-19. doi: 10.1128/mBio.02834-19 (PMC6974565; doi:10.1128/mBio.02834-19)
Supplement: TABLE S1 [file mBio.02834-19-st001.docx]

**Table S1.** Effects of neurotrophic factors on electrophysiological parameters of human ileal mucosa mounted on Ussing chambers.

Comparisons were done with two-way ANOVA followed by Tuckey’s multiple comparison test and values are shown as median (25th-75th percentile). No differences were found between treatments from time 0 min to 120 min.

| Parameter | Control | | | GSNO | | | GDNF | | |
| --- | --- | --- | --- | --- | --- | --- | --- | --- | --- |
|  | 0 min | 60 min | 120 min | 0 min | 60 min | 120 min | 0 min | 60 min | 120 min |
| TER Ω.cm^2^ | 78.6 (67.4-85.8) | 68.6 (57.5-69.8) | 59.3 (50.8-62.7) | 75.7 (60.9-83.8) | 66.6 (51.9-77.4) | 58.2 (47.6-75.4) | 90.9 (68.4-96.8) | 83.0 (59.4-87.1) | 75.1 (51.5-84-2) |
| PD mV/cm^2^ | -0.9 (-2.2- -0.6) | -0.5 (-1.6- -0.1) | -0.5 (-1.7- -0.2) | -1.5 (-2.5- -0.9) | -0.9 (-1.7- -0.5) | -1.0 (-1.6- -0.4) | -0.8 (-1.0- -0.6) | -0.4 (-0.6- -0.1) | -0.5 (-0.8- -0.4) |
| Isc µA/cm^2^ | 11.5 (8.0-28.0) | 7.7 (2.5-25.1) | 9.1 (3.2-29.2) | 18.7 (13.3-37.4) | 13.3 (7.3-31.1) | 17.2 (5.9-32.0) | 9.5 (6.3-13.3) | 5.3 (0.8-9.1) | 7.1 (5.3-12.2) |
