# Supplementary material for: Neurotrophic Factors Protect the Intestinal Barrier from Rotavirus Insult in Mice
Source: mBio. 2020 Jan 21;11(1):e02834-19. doi: 10.1128/mBio.02834-19 (PMC6974565; doi:10.1128/mBio.02834-19)
Supplement: TABLE S2 [file mBio.02834-19-st002.docx]

**Table S2.** Effects of neurotrophic factors on electrophysiological parameters of mice ileal mucosa mounted on Ussing chambers.

| Parameter | Control | | | GSNO | | | GDNF | | |
| --- | --- | --- | --- | --- | --- | --- | --- | --- | --- |
|  | 0 min | 60 min | 120 min | 0 min | 60 min | 120 min | 0 min | 60 min | 120 min |
| TER Ω.cm^2^ | 51.8 (40.6-67.9) | 47.2 (31.9-65.2) | 40.5 (27.2-59.7) | 46.5 (31.3-95.8) | 41.5 (28.7-84.7) | 38.4 (26.0-74.4) | 62.3 (42.2-73.3) | 65.5 (36.8-76.8) | 63.1 (33.6-79.5) |
| PD mV/cm^2^ | -0.4 (-0.6- -0.2) | -0.5 (-0.7- -0.3) | -0.5 (-0.9- -0.4) | -0.6 (-1.1- -0.3) | -0.7 (-1.2- -0.3) | -0.7 (-0.9- -0.3) | -0.6 (-0.9- -0.3) | -0.5 (-1.0- -0.2) | -0.4 (-0.9- -0.3) |
| Isc µA/cm^2^ | 5.8 (4.8-12.8) | 13.0 (5.1-18.1) | 21.6 (5.4-25.7) | 12.7 (5.7-25.3) | 15.2 (5.7-31.4) | 14.6 (8.7-36.2) | 8.7 (5.4-20.0) | 7.9 (4.3-23.7) | 7.0 (4.4-22.8) |

Comparisons were done with two-way ANOVA followed by Tuckey’s multiple comparison test and values are shown as median (25th-75th percentile). No differences were found between treatments from time 0 min to 120 min.
