# Supplementary material for: Neurotrophic Factors Protect the Intestinal Barrier from Rotavirus Insult in Mice
Source: mBio. 2020 Jan 21;11(1):e02834-19. doi: 10.1128/mBio.02834-19 (PMC6974565; doi:10.1128/mBio.02834-19)
Supplement: TABLE S3 [file mBio.02834-19-st003.docx]

**Table S3.** Electrophysiological parameters of rotavirus-infected ileal mucosa of mice, mounted on Ussing chambers.

| Parameter | Non-infected | | | Rotavirus-infected | | |
| --- | --- | --- | --- | --- | --- | --- |
|  | 0 min | 60 min | 120 min | 0 min | 60 min | 120 min |
| TER Ω.cm^2^ | 51.1 (27.8-64.1) | 45.9 (25.1-62.6) | 35.6 (21.5-51.8) | 63.8 (38.9-69.7) | 53.8 (35.6-63.6) | 42.3 (28.5-46.5) |
| PD mV/cm^2^ | -0.9 (-1.5- -0.5) | -0.6 (-1.4- -0.1) | -0.4 (-1.2- -0.2) | -0.5 (-0.7- -0.2) | -0.3 (-0.6- -0.2) | -0.3 (-0.4- -0.1) |
| Isc µA/cm^2^ | 16.9 (8.7-20.2) | 15.2 (5.5-23.7) | 14.5 (6.5-19.2) | 6.2 (4.4-12.9) | 9.0 (2.1-9.8) | 7.8 (2.5-10.6) |

Comparisons were done with two-way ANOVA followed by Tuckey’s multiple comparison test and values are shown as median (25th-75th percentile). No differences were found between treatments from time 0 min to 120 min.
